# Attitudes towards Safe Listening Measures in Entertainment Venues: Results from an International Survey among Young Venue-Goers

**DOI:** 10.3390/ijerph182312860

**Published:** 2021-12-06

**Authors:** Nicola Diviani, Shelly Chadha, Malachi Ochieng Arunda, Sara Rubinelli

**Affiliations:** 1 Person-Centered Health Care and Health Communication Group, Swiss Paraplegic Research, Guido A. Zäch Strasse 4, 6207 Nottwil, Switzerland; sara.rubinelli@paraplegie.ch; 2 Department of Health Sciences and Medicine, University of Lucerne, 6002 Lucerne, Switzerland; 3 Department for Noncommunicable Diseases, World Health Organization (WHO), 1202 Geneva, Switzerland; chadhas@who.int (S.C.); arundam@who.int (M.O.A.)

**Keywords:** hearing loss, recreational, public venues, entertainment venues, survey

## Abstract

Background: Sustained exposure to excess noise in recreational settings is among the main causes of hearing loss among young adults worldwide. Within a global effort to develop standards for safe listening in entertainment venues, this study aims at identifying modifiable factors (knowledge, attitudes, and beliefs), which can hinder or facilitate the acceptance of safe listening measures in public venues among young venue-goers. Methods: An online questionnaire was developed inspired by the Health Belief Model. It was divided into five sections: (i) socio-demographics (ii) listening habits, (iii) experiences with loud music, (iv) knowledge, attitudes, and beliefs, and (v) willingness to change. Participants were recruited through social media. Results: 2264 individuals aged 16–35 completed the questionnaire. Most visited entertainment venues relatively infrequently, with the majority of them only visiting once per month or less. Nevertheless, most reported having experienced the negative consequences of listening to loud music. Overall, most people were favorable towards preventive measures, especially quiet areas. Conclusion: Our findings stress the urge to address the issue of safe listening in public venues and support an approach based on the introduction of standards. Moreover, they provide us with information on key factors to be considered when introducing and communicating preventive measures in public entertainment venues.

## 1. Introduction

Around 5% people worldwide live with hearing loss and estimates suggest that around one in four will be living with some degree of hearing loss by 2050 [1,2]. The impact of hearing loss is broad and can be profound. It includes a loss of the ability to communicate with others, delayed language development in children, which can lead to social isolation, loneliness, and frustration, particularly among older people with hearing loss [3,4]. Many areas lack sufficient accommodations for hearing loss, which affect academic performance and options for employment [5,6]. Children with hearing loss and deafness in developing countries rarely receive any schooling. The World Health Organization (WHO) estimates that unaddressed hearing loss costs the global economy USD 980 billion annually due to health sector costs (excluding the cost of hearing devices), costs of educational support, loss of productivity, and societal costs [2]. Excessive noise is among the leading causes of acquired hearing loss and it is increasingly encountered in many aspects of day-to-day life [7]. Among the many people exposed to noise at their place of work, for instance, occupational hearing loss is common [8,9], and the cost of compensation is extremely high [8,10]. Additionally, concern is growing about the ever-greater exposure to noise in recreational settings; unsafe levels of sound are frequently experienced in a variety of non-occupational settings such as nightclubs, discotheques, pubs, bars, cinemas, concerts, live sporting events, and even fitness classes [11]. Moreover, recreational devices such as personal music players and video game consoles that emit sounds are commonly operated at unsafe volumes [11].

The WHO estimates that 1.1 billion young people worldwide could be at risk of hearing loss due to unsafe listening practices [1,11]. Nearly half of all teenagers and young adults (12–35 years old) in middle- and high-income countries are exposed to unsafe levels of sound from the use of personal audio devices and some 40% of them are exposed to potentially damaging sound levels at clubs, discotheques, and bars [11]. 

There are several actions that can be taken to control the detrimental effects of too high or unsafe sound levels in public entertainment venues, for instance providing customers with hearing protection, ensuring venues have quiet areas where people can rest their ears, or the introduction of volume limits. Yet, clear rules for safe listening venues are only in place in a few countries worldwide. To address this gap, the WHO is working with experts and a wide range of stakeholders to develop a standard for safe listening in entertainment venues [12,13]. If applied, such standards would greatly contribute to protecting hearing. In line with what was completed for the standard for personal listening devices [14], the standard will include a more technical part about the definition of safe sound levels and about ways to ensure that it is met, but there will also be a section with recommendations on how the different interventions can and should be communicated to the end users, i.e., the clients of venues. 

There is a large body of evidence on the technical aspects, including the safe limits in terms of volume and length of exposure, how sound travels across different rooms, or what technical solutions exist to mitigate possible detrimental effects (see, e.g., [15]). Less is known about possible individual barriers to the acceptance of such interventions and about how they can be addressed through communication. So far, studies on attitudes and beliefs towards hearing protection have focused mostly on personal listening devices [16,17,18,19], while only a few studies have investigated factors associated with attitudes towards preventive measures in venues [20].

Against this background, and within the overall objective of informing the development of standards for safe listening at entertainment venues, this paper aims at identifying modifiable factors (knowledge, attitudes, and beliefs), which can hinder or facilitate the acceptance of safe listening practices in public venues among young venue-goers. 

## 2. Methods

### 2.1. Recruitment and Sample

Data were collected between March and October 2020 through an online survey shared on WHO’s social media platforms. Additionally, the link to the survey was sent to key stakeholders in the field of ear and hearing health worldwide, who were asked to share it in their local communities. To be eligible to take part in the survey, participants had to have a good command of English, French, Spanish, Russian, or Chinese, be aged between 16 and 35, and have visited an entertainment venue (bar, club, disco, concert hall, gym, or festival) more than once per year. To ensure that participants completed the survey with due diligence, we assessed survey response time and excluded those participants who completed the survey in less than five minutes. A total of 2264 individuals completed the questionnaire. 

### 2.2. Instrument and Measures

An online questionnaire was developed partly inspired by the Health Belief Model (HBM) [21], as well as building on findings from past empirical studies in the context of music- and noise- induced hearing loss (see, e.g., [22]). According to the HBM, people’s beliefs about health problems, perceived benefits of action, and barriers to action explain attitudes towards and engagement in health-promoting behaviors [23]. The model was chosen as a reference as its constructs have proven to be relevant predictors of acceptance of preventive measures across health-related issues, including in the field of hearing protection [19,22]. The questionnaire was developed in English and subsequently translated into French, Spanish, Russian, and Chinese. It was divided into five main sections that assessed individuals’ (i) socio-demographics (ii) listening habits in venues, (iii) experiences with loud music in public venues, (iv) knowledge, attitudes, and beliefs about safe listening in public venues, and (v) willingness to change. The full questionnaire is available as Supplementary Materials (S1).

#### 2.2.1. Socio-Demographics

Data were collected about the respondents’ gender, age, educational level, and country of residence.

#### 2.2.2. Listening Habits in Venues

Respondents were asked through a single-item question to indicate the frequency of visits in public venues with music, including bars, clubs, discos, concert halls, gyms, and festivals (1 = “Several times per year”, 2 = “Once per month”, 3 = “Several times per month”, 4 = “Once per week”, 5 = “Several times per week”, 6 = “Daily”). A dichotomous variable was created to be used in bivariate analyses, with 0 = “Less than once per week” and 1 = “Once per week or more”).

#### 2.2.3. Experience with Loud Music in Public Venues

In this section of the questionnaire participants were asked to rate their subjective perception of the loudness of music in each of the venues mentioned above on a 5-point scale (1 = “Too high”, 5 = “Too low”). In addition, they were asked about the frequency they engage in four preventive behaviors when the sound is too high: leave the venue, wear hearing protection (e.g., earplugs), search for a quiet area, and ask to reduce the volume. Respondents were asked to answer on a 5-point scale (1 = “Always”, 5 = “Never”). Finally, participants were asked to indicate whether they had any experience of tinnitus after exposure to loud sounds in public venues. Answer options were on a 5-point scale (1 = “Always”, 5 = “Never”).

#### 2.2.4. Knowledge, Attitudes, and Beliefs

To assess knowledge about hearing loss, participants were asked to evaluate the statement “Listening to sounds above 80 decibels over a period of time can cause permanent damage to your hearing.” on a 5-point scale (1 = “Definitely false”, 5 = “Definitely true”). Answers were subsequently recoded into a dichotomous variable (1, 2, and 3 = “Incorrect” and 4 and 5 = “Correct”). Attitudes towards preventive measures in venues were assessed with 5 items asking respondents to rate their favorableness towards five different preventive measures: distribution of hearing protection (e.g., earplugs); quiet zones; volume limits; informational/awareness material (e.g., leaflets and posters); warnings (e.g., a flashing light when music reaches a certain level). Answers were on a five-point scale (1 = “Not at all favorable”, 5 = “Completely favorable”). For analytical purposes, an average score to indicate the overall attitude was subsequently computed (Cronbach’s α = 0.838; M = 3.39, SD = 1.15). Beliefs were assessed by asking respondents to rate their agreement with 12 statements about different aspects of hearing protection in public venues (e.g., “I think earplugs are uncomfortable” or “I would like to be notified when the sound in the entertainment venue is too loud” on a five-point scale (1 = “Completely disagree”, 5 = “Completely agree”). In this context, we also assessed perceived susceptibility (seven-point scale, 1 = “Very low chance”, 7 = Very high chance) to hearing loss following listening to loud music in public venues and perceived severity of hearing loss (seven-point scale, 1 = “Not at all disruptive”, 7 = “Very disruptive”) through two single-items. 

#### 2.2.5. Willingness to Change Behavior

Respondents were asked whether they would be willing to change their listening habits in entertainment venues, if they were more informed about the risks of listening to loud music for a long time. Response options were on a 7-point scale (1 = “Strongly disagree”; 7 = “Strongly agree”).

### 2.3. Data Analysis

Descriptive statistics (frequencies, means) were used to describe the sample in terms of socio-demographics and other relevant characteristics. Group differences in the different aspects were assessed using one-way ANOVAs. The association of the variables of interest with attitudes towards preventive measures was assessed using linear regression analyses. Statistical analysis was conducted using IBM SPSS 26 (IBM, Armonk, New York, NY, USA).

## 3. Results

### 3.1. Sample Characteristics and Frequency of Visits to Venues

Survey respondents were predominantly females (65%, n = 1483) and were on average 24 years old (range: 16–35; SD = 4.8). Most commonly, respondents had a college degree (36.8%, n = 834) or a high school degree (24.9%, n = 563). Only a few had less than a high school degree or less (3%, n = 68). The sample included individuals from more than 40 countries. Most respondents were from countries within the African region (46.7%, n = 1057), followed by the Western Pacific Region (23.9%, n = 541). Only a few respondents were from the Eastern Mediterranean region (0.2%, n = 4). The majority of respondents reported visiting an entertainment venue once a month or less (56%, n = 1065). A more detailed overview of the respondents’ socio-demographic characteristics and frequency of visits in venues can be found in Table 1.

### 3.2. Experience with Loud Music in Public Venues

In general, respondents reported perceiving the sound level in venues as somehow too high. This was particularly true for discos (M = 1.68, SD = 0.96) and clubs (M = 1.64, SD = 0.92). Overall, the sound level in gyms was rated as right (M = 3.00, SD = 1.03). Female respondents rated the sound level in bars (*p* < 0.05), discos (*p* < 0.05), clubs (*p* < 0.01), and concert halls (*p* < 0.05) as higher compared to males. Older age was associated with perceiving the sound as higher in all venues (*p* < 0.001) except for festivals. Those who visit venues less frequently rated the sound level in bars (*p* < 0.001) and gyms (*p* < 0.01) as higher compared to more frequent visitors. Lastly, those with a higher educational level rated the sound levels in bars, clubs, discos, and gyms as higher (*p* < 0.001) compared to those with lower education.

As regards preventive measures adopted, the most commonly mentioned was searching for a quieter area, with 90.9% of the sample reporting to have done this at least once, followed by leaving the venue (82.0%). Less frequently mentioned were asking to reduce the volume (44.3%) and using hearing protection (39.0%). Female respondents were slightly more likely to have used hearing protection (*p* < 0.05), and those who visit venues less were more likely to have left the venue because of high sound levels (*p* < 0.01). Those with higher educational level (*p* < 0.05) and older respondents (*p* < 0.001) were more likely to have adopted all the preventive measures.

When it comes to the consequences of being exposed to loud sounds, more than 80% of the respondents reported having experienced tinnitus at least once. Around 20% of respondents even reported experiencing it always or often. Those with a lower educational level and those visiting venues more often were more likely to have experienced tinnitus (*p* < 0.05).

### 3.3. Knowledge, Attitudes, and Beliefs

More than 80% of respondents (81.1%, n = 1836) answered correctly the question about the impact of loud music on hearing, with lower percentages of correct answers among younger respondents and among those with a lower educational level (*p* < 0.001). 

Overall, respondents reported a somewhat favorable attitude towards the different preventive measures. The measure evaluated most positively was the introduction of quiet zones (M = 3.86, SD = 1.36), while the one receiving least consensus was the distribution of hearing protection (M = 2.97, SD = 1.60). Female respondents were shown to be more favorable to all preventive measures (*p* < 0.05). Those with a higher educational level had more favorable attitudes towards the introduction of volume limits (*p* < 0.001), while those with a lower education were more favorable towards quiet zones (*p* < 0.01). Younger respondents were less favorable towards volume limits but more favorable towards the introduction of quiet zones (*p* < 0.001). No significant differences were found for frequent and less frequent venue-goers.

As regards beliefs, respondents overall tended to agree more with beliefs favoring preventive measures, in particular as regards the introduction of quiet zones, and to disagree with the ones against preventive measures (see Table 2 for more details). One exception were beliefs about earplugs and hearing protection, which were perceived as uncomfortable by almost half of the respondents and as an interference to the enjoyment of music by more than two-thirds. Overall, female respondents, older respondents, those with a higher educational level, and those who visit venues less frequently were shown to hold more favorable beliefs about volume limits, quiet zones, and notifications. 

Regarding risk perception, we observed that, overall, the respondents reported relatively high perceived susceptibility to hearing loss due to listening to high volume music (M = 5.20, SD = 1.7) and an even higher perceived severity of hearing loss (M = 6.32, SD = 1.3). Female respondents showed higher levels of both perceived susceptibility (*p* < 0.01) and perceived severity (*p* < 0.001). Those with a higher educational level perceived hearing loss as less disruptive (*p* < 0.05). Frequent venues goers (*p* < 0.05) and older respondents (*p* < 0.001) reported higher perceived susceptibility.

### 3.4. Willingness to Change Behavior

Most respondents (61.4% 9 expressed at least some intention to change behavior. Female respondents (*p* < 0.01), younger respondents (*p* < 0.001), those with a lower educational level (*p* < 0.001), and more frequent venue goers (*p* < 0.001) all showed a higher intention. 

### 3.5. Predictors of Attitudes towards Preventive Measures

A series of linear regression analyses showed that controlling for gender, age, education, and frequency of visits, a number of factors were significantly associated with a more positive attitude towards preventive measures. In particular, as shown in Table 3, more positive attitudes towards preventive measures were observed among people who perceive the sound level as too loud in clubs, those who have experienced tinnitus more frequently, those holding more favorable beliefs towards measures, those who perceive themselves more at risk (both perceived severity and susceptibility), and those who are more willing to change. On the other hand, those experiencing the sound as too loud in bars and gyms and those holding more negative beliefs towards safe listening practices were less favorable to the measures.

## 4. Discussion 

This study aimed at identifying modifiable factors associated with attitudes towards preventive measures in public entertainment venues, which might hinder or facilitate the acceptance of safe listening options among younger patrons. Our findings showed that most participants visit entertainment venues relatively infrequently, with the majority of them only visiting once per month or less. Nevertheless, most of them reported having first-hand experience of the consequences of listening to loud music, with more than 80% reporting having experienced tinnitus at least once and almost 20% experiencing it often or every time after visiting a venue. In general, respondents reported perceiving the sound level in venues as somehow too high and, especially for clubs, this was associated with more favorable attitudes towards preventive measure. Our analyses also showed very clearly that, overall, people in our sample were favorable towards preventive measures, with most of them holding positive beliefs toward safe listening practices such as spending time in quiet areas or being notified when the sound level is too high, and being willing to change their behavior. Overall, these findings have important implications: first, they stress the urge to address the issue of safe listening in public venues; second, they support an approach based on the introduction of standards, with clear rules to which venues have to adhere. 

Aside from these general considerations, our findings also provide us with some important information on key factors to be taken into account and addressed when introducing preventive measures in public entertainment venues. A first point regards differences in the acceptance of different types of safe listening measures. Quiet areas were by far the preferred measure; most respondents agreed with the importance of having a place where they can rest their ears in entertainment venues, and only a few did not see the point of introducing them. Even though to a slightly lesser extent, provision of information on safe listening and notifications when the sound level is too high for a long time were overall perceived favorably. A less clear picture emerges regarding the introduction of volume limits. More than half of the respondents declared that they would feel safer with the introduction of volume limits, and around one third stated that entertainment venues should be free to decide at which level to play music. Finally, hearing protection turned out to be the least favorite measure, with around 70% of the respondents being concerned that earplugs would interfere with their enjoyment of music and around half of them saying that they are uncomfortable. Only 30% of respondents reported that they would be willing to pay for hearing protection. This result is in line with other studies in the field [19,24]. In light of these findings, we suggest that the introduction of quiet areas, where technically possible, should be prioritized as it would encounter less resistance compared to other measures. Quiet areas would also be an ideal place to provide patrons with detailed information about the dangers of listening to loud music and on ways to prevent long-term consequences. This could be a first step for raising awareness and setting the stage for implementing less widely accepted measures. 

A second point regards the development of communication measures to accompany the introduction of preventive measures in public entertainment venues. Overall, our analyses did not point to a single specific factor, or group of factors, playing a prominent role in explaining differences in attitudes. Nevertheless, our findings highlighted some group differences in beliefs and attitudes that can inform the definition of the target audience and the contents of future communication interventions.

A first set of considerations regards the *target audience*. We observed that, in line with studies conducted in related fields [25,26], both being male and having a lower educational level were associated with a less favorable overall attitude towards preventive measures. It is therefore important that these factors are accounted for when planning a communication intervention. This could mean, for instance, making sure to include appeals of framing techniques that are known to be particularly persuasive among males, such as messages with a positive rational appeal (vs. a negatively framed emotional one) [27] and to make sure to use simple messages in plain language that can easily be understood also by people with a lower educational level [28,29]. 

A second set of considerations regards the actual *contents* of a possible communication intervention. We observed that past experience of tinnitus is linked to more positive attitudes towards preventive measures. Communication should therefore emphasize and make more salient the experience of tinnitus, for instance by making people experience in first person how living with a constant ringing in their ears would look like. Virtual reality has been proven to be an effective tool for this purpose in other fields, and could therefore be considered [30,31]. We also observed that perceived susceptibility to hearing loss and perceived severity of hearing loss were associated with more positive attitudes towards measures. In light of this, communication interventions should focus on increasing risk perception. Effective strategies in this context include, for instance, using narrative communication and storytelling techniques to share personal stories of people that are perceived as belonging to the same group (i.e., young people visiting venues) [32]. Finally, as mentioned earlier, we observed that some preventive measures are less favored than others and would therefore need a stronger persuasion effort. We refer here in particular to hearing protection, which is perceived by many as uncomfortable and as an interference with music. When promoting this practice, in addition to making sure that it is offered for free, it is therefore central that these barriers are addressed explicitly and confuted by sources with a high degree of credibility (i.e., that is perceived as competent, trustworthy, and attractive) among the target audience. An example in this context could be a famous musician or DJ saying that not all earplugs affect the quality of the music experience [33].

We acknowledge that the study has a number of limitations. First, data were collected through a self-administered anonymous online survey and thus relied on self-reporting rather than objective measures. Moreover, the questionnaire, although built from adapting existing tools, was not validated. Second, the cross-sectional study design we used does not provide conclusive evidence with regard to the directions of relationships in the data. Lastly, the use of a self-selected sample recruited through social media channels limits the generalizability of our descriptive findings. In particular, our respondents might be more interested in the topic of safe listening compared to the general public and therefore more favorable towards preventive measures. Our results, however, are in line with those of past research in the field, thus increasing our confidence in their validity. 

## 5. Conclusions

Sustained exposure to excess noise in recreational settings is among the leading causes of acquired hearing loss among young adults worldwide. This is why the WHO is bringing together health authorities and policy makers from across the globe to tackle this issue by developing a standard for safe listening in public entertainment venues. Like any other public health intervention, however, the success of the introduction of such a standard largely depends on the extent to which these relative measures are accepted by the public. This study, conducted among young venue-goers from a variety of countries worldwide, shows that, overall, patrons hold a positive attitude towards the introduction of safe listening practices in public entertainment venues, suggesting that that there is indeed a need for such measures Additionally, the study highlighted some possible factors, which could hinder their acceptance and, therefore, need to be addressed. These factors include socio-demographic characteristics, such as being a male or having a lower level of education, as well as some personal factors, such as having low risk perception or perceiving barriers to the adoption of the measures. The findings of this study support the idea that a communication intervention, specifically targeted to those who are more likely to be resistant, addressing the main concerns, and increasing the risk perception of hearing loss, holds great potential to facilitate the acceptance of a standard and of the relative preventive measures.

## Figures and Tables

**Table 1:** Sample characteristics.

	Mean	SD	n	%
**Gender**				
Male			747	33.0
Female			1483	65.5
Other			7	0.3
I prefer not to say			27	1.2
**Age**	23.8	4.8		
**Educational level**				
Less than High School			68	3.0
High School/GED			563	24.9
Some college			282	12.5
2-year College Degree			163	7.2
4-year College Degree			671	29.6
Master’s Degree			283	12.5
Doctoral Degree			45	2.0
Professional Degree (JD, MD)			119	5.3
I prefer not to say			70	3.1
**Region**				
African Region (AFRO)			1057	46.7
Region of the Americas (PAHO)			211	9.3
South-East Asia Region (SEARO)			102	4.5
European Region (EURO)			346	15.3
Eastern Mediterranean Region (EMRO)			4	0.2
Western Pacific Region (WPRO)			541	23.9
I prefer not to say			**3**	**0.1**
**Frequency of visits to entertainment venues**				
Several times a year			1057	46.7
Once a month			211	9.3
Several times a month			102	4.5
Once a week			346	15.3
Several times a week			4	0.2
Daily			541	23.9

**Table 2:** Beliefs about preventive measures.

	% Somewhat or Completely Agree								
	All	Gender		Educational Level ^a^		Age		Frequency of Visits ^b^	
		Male	Female	Low	High	<25	25+	Low	High
*I would appreciate having a place within the entertainment venue where I can rest my ears*	81.6%	79.5%	82.9%	82.9%	81.7%	**82.9%**	**78.5%**	81.1%	83.3%
*Entertainment venues should provide information about the risks of listening to loud music and how to protect hearing*	70.0%	**68.1%**	**71.2%**	**65.1%**	**74.2%**	68.9%	72.7%	70.4%	68.4%
*I would like to be notified when the sound in the entertainment venue is too loud*	66.7%	64.5%	68.2%	**61.1%**	**71.3%**	65.6%	69.3%	67.1%	64.9%
*If a law existed limiting the volume in entertainment venues I would feel safer*	56.7%	**50.3%**	**59.3%**	**47.2%**	**64%**	**53.3%**	**64.9%**	**57.8%**	**52.2%**
I think that earplugs are uncomfortable	49.2%	**46.2%**	**50.7%**	48.5%	50.4%	48.8%	49.9%	**48.1%**	**53.3%**
I would not pay attention to informational materials when I am visiting an entertainment venue	32.8%	34.7%	32.1%	**38.2%**	**29.7%**	**34.9%**	**27.8%**	**31.4%**	**38.7%**
*I would not mind paying for hearing protection (e.g., for earplugs)*	30.2%	29.2%	31%	**27.3%**	**32.4%**	29%	33%	30.8%	27.8%
*Using earplugs does not interfere with my enjoyment of music*	27.6%	27%	28.2%	**24.9%**	**29.6%**	26.7%	30%	27.6%	28%
Entertainment venues should be free to decide at which level to play music	26.1%	29.6%	24.2%	**32.5%**	**21.9%**	**68.9%**	**72.7%**	**25%**	**30.4%**
I would be annoyed by being notified when the sound level is too high	20.2%	**22.5%**	**18.9%**	**23.5%**	**17.9%**	21.2%	17.6%	**19.2%**	**23.8%**
I would spend time in the quiet zone only if I got something in exchange (e.g., a free drink)	20.2%	20.7%	19.9%	**22.3%**	**18.4%**	21%	18.1%	**19.2%**	**23.8%**
I do not see the need of having a quiet zone in an entertainment venue	12.2%	14.1%	11.5%	11.6%	12.8%	**11.3%**	**14.6%**	11.9%	13.8%

Notes: Response options are on a 5-point scale: 1 = ‘Completely disagree; 5 = ‘Completely agree’. Statements in *italics* are in favor of safe listening practices. Percentages in **bold** indicate statistically significant differences (*p* < 0.05). ^a^ Low = Less than a college degree, High = College degree or higher; ^b^ Low = Less than once per week, High = Once per week or more.

**Table 3:** Predictors of attitudes towards preventive measures.

Predictors	Attitude towards Preventive Measures
	B (95% CI)
**Perceived sound level too loud in**	
Bars	0.228 (0.113 to 0.342) **
Clubs	−0.175 (−0.332 to −0.018) *
Discos	0.016 (−0.118 to 0.149)
Concert halls	0.009 (−0.099 to 0.118)
Gyms	0.184 (0.088 to 0.281) **
Festivals	−0.055 (−0.176 to 0.065)
**Experience of tinnitus**	−0.091 (−0.137 to −0.045) **
**Knowledge**	0.002 (−0.123 to 0.127)
**Beliefs**	
I would appreciate having a place within the entertainment venue where I can rest my ears	0.220 (0.168 to 0.263) **
Entertainment venues should provide information about the risks of listening to loud music and how to protect hearing	0.129 (0.079 to 0.179) **
I would like to be notified when the sound in the entertainment venue is too loud	0.134 (0.082 to 0.186) **
If a law existed limiting the volume in entertainment venues I would feel safer	0.042 (−0.003 to 0.086)
I think that earplugs are uncomfortable	−0.043 (−0.080 to −0.006) *
I would not pay attention to informational materials when I am visiting an entertainment venue	−0.008 (−0.043 to 0.028)
I would not mind paying for hearing protection (e.g., for earplugs)	0.011 (−0.022 to 0.044)
Using earplugs does not interfere with my enjoyment of music	0.094 (0.060 to 0.129) **
Entertainment venues should be free to decide at which level to play music	−0.045 (−0.0081 to −0.009) *
I would be annoyed by being notified when the sound level is too high	−0.079 (−0.117 to −0.042) **
I would spend time in the quiet zone only if I got something in exchange (e.g., a free drink)	−0.009 (−0.042 to 0.024)
I do not see the need of having a quiet zone in an entertainment venue	−0.047 (−0.090 to −0.003) *
**Perceived susceptibility**	0.110 (0.081 to 0.138) **
**Perceived severity**	0.127 (0.090 to 0.163) **
**Willingness to change**	0.128 (0.105 to 0.150) **

Notes: Separate models were calculated for each construct under investigation. * *p* < 0.05; ** *p* < 0.001. Models are adjusted for gender, age, education, and frequency of visits to entertainment venues.

## Data Availability

The data presented in this study are available on reasonable request from the corresponding author.

## Supplementary Materials

The following is available online at https://www.mdpi.com/article/10.3390/ijerph182312860/s1, the questionnaire of the online survey on safe listening in entertainment venues.
