# Supplementary material for: Attitudes towards Safe Listening Measures in Entertainment Venues: Results from an International Survey among Young Venue-Goers
Source: Int J Environ Res Public Health. 2021 Dec 6;18(23):12860. doi: 10.3390/ijerph182312860 (PMC8657835; doi:10.3390/ijerph182312860)
Supplement: Supplementary file 1 [file ijerph-18-12860-s001.zip › ijerph-1470924-supplementary.pdf]

World Health Organization

Make Listening Safe

Survey on safe listening in entertainment venues

Thank you very much for your interest in participating in this survey.

The present survey is carried out by the World Health Organization (Geneva, Switzerland). Its objective is to investigate how people listen to music in entertainment venues (e.g., bars, clubs, concert halls, gyms, festivals) and their attitudes towards preventive measures and regulations in this context.

Completing the questionnaire will require about 5-7 minutes. Your participation to this survey is voluntary. You have the right to withdraw your participation at any time, without giving a reason, by simply closing the window of your browser. The results of this survey will be used in a completely anonymous manner.

For any question related to the survey or to your participation you can contact the study responsible Dr. Nicola Diviani at the University of Lucerne: Nicola.Diviani@unilu.ch.

ELECTRONIC CONSENT: Please confirm that you have read the information above and that you voluntarily agree to take part to the survey. *

( ) I confirm that I have read the information above and that I voluntarily agree to take part to the survey

---

1. How often do you **visit entertainment venues** (e.g., bars, clubs, festivals, concert halls, gyms)?

- ◯ Never
- ◯ Less than once a year
- ◯ Once a year
- ◯ Several times a year
- ◯ Once a month
- ◯ Several times a month
- ◯ Once a week
- ◯ Several times a week
- ◯ Daily

World Health Organization

Make Listening Safe

2. For each of the entertainment venues listed below, please indicate **how often do you visit them**.

	Less than once a year	Once a year	Several times a year	Once a month	Several times a month	Once a week	Several times a week	Daily	I do not visit this venue
Bars	○	○	○	○	○	○	○	○	○
Clubs	○	○	○	○	○	○	○	○	○
Discos	○	○	○	○	○	○	○	○	○
Concert halls	○	○	○	○	○	○	○	○	○
Gyms	○	○	○	○	○	○	○	○	○
Festivals	○	○	○	○	○	○	○	○	○

3. For each of the entertainment venues listed below, please indicate **<u>how much time do you usually spend in the venue on a single visit</u>**.

	Less than an hour	1 -3 hours	More than 3 hours	I do not visit this venue
Bars				
Clubs				
Discos				
Concert halls				
Gyms				
Festivals				

4. For each of the entertainment venues listed below, please indicate for **<u>how many years have you kept this routine</u>**.

	Less than one year	1 -3 years	More than 3 years	I do not visit this venue
Bars				
Clubs				
Discos				
Concert halls				
Gyms				
Festivals				

World Health Organization

Make Listening Safe

5. How would you rate the **overall sound level** in each entertainment venue?

	1. Too high	2	3	4	5. Too low	I do not visit this venue
Bars	○	○	○	○	○	○
Clubs	○	○	○	○	○	○
Discos	○	○	○	○	○	○
Concert halls	○	○	○	○	○	○
Gyms	○	○	○	○	○	○
Festivals	○	○	○	○	○	○

6. When you are in an entertainment venue and the volume of the music is too loud, **how often have you taken one of the following actions**?

	Always	Often	Sometimes	Rarely	Never
Leave the venue	○	○	○	○	○
Wear hearing protection (e.g., earplugs)	○	○	○	○	○
Search for a quieter area	○	○	○	○	○
Ask to reduce the volume	○	○	○	○	○

7. How often have you **experienced having tinnitus (ear ringing or buzzing) after visiting** an entertainment venue?

- ◯ Never
- ◯ Rarely
- ◯ Sometimes
- ◯ Often
- ◯ Every time

8. In general, **<u>how favorable are you of the following preventive measures in entertainment venues</u>**?

	1 Not at all	2	3	4	5 Completely
Distribution of hearing protection (e.g., earplugs)	○	○	○	○	○
Quiet zones	○	○	○	○	○
Volume limits	○	○	○	○	○
Informational/Awareness material (e.g., leaflets, posters)	○	○	○	○	○
Warnings (e.g., flashing light when music reaches a certain level)	○	○	○	○	○

9. Please rate your **<u>agreement with the following statements</u>**.

World Health Organization

Make Listening Safe

	Completely disagree	Somewhat disagree	Neither agree nor disagree	Somewhat agree	Completely agree
I think that earplugs are uncomfortable	○	○	○	○	○
I would appreciate having a place within the entertainment venue where I can rest my ears	○	○	○	○	○
Entertainment venues should provide information about the risks of listening to loud music and how to protect hearing	○	○	○	○	○
If a law existed limiting the volume in entertainment venues I would feel safer	○	○	○	○	○
I would like to be notified when the sound in the entertainment venue is too loud	○	○	○	○	○
Using earplugs does not interfere with my enjoyment of music	○	○	○	○	○
I do not see the need of having a quite zone in an entertainment venue	○	○	○	○	○

World Health Organization

Make Listening Safe

I would not pay attention to informational materials when I am visiting an entertainment venue	○	○	○	○	○
Entertainment venues should be free to decide at which level to play music	○	○	○	○	○
I would be annoyed by being notified when the sound level is too high	○	○	○	○	○
I would not mind having to pay for hearing protection (e.g., for earplugs)	○	○	○	○	○
I would spend time in the quite zone only if I get something in exchange (e.g., a free drink)	○	○	○	○	○

10. True or false? Listening to sounds **above 80 decibels over a period of time** can cause **permanent damage** to your hearing.

- ○ Definitely true
- ○ Probably true
- ○ Probably false
- ○ Definitely false
- ○ I don't know

11. What is the chance that you will experience hearing loss from listening to loud music?

- ◯ 1 Very low chance
- ◯ 2
- ◯ 3
- ◯ 4
- ◯ 5
- ◯ 6
- ◯ 7 Very high chance

12. Overall, how disruptive would hearing loss be in your life?

- ◯ 1 Not at all disruptive
- ◯ 2
- ◯ 3
- ◯ 4
- ◯ 5
- ◯ 6
- ◯ 7 Extremely disruptive

13. If the World Health Organisation (WHO) informed me about the risks of listening to loud music for a long time, I would be willing to change my listening habits in entertainment venues.

- ◯ 1 Strongly disagree
- ◯ 2
- ◯ 3
- ◯ 4
- ◯ 5
- ◯ 6
- ◯ 7 Strongly agree

World Health Organization

Make Listening Safe

To conclude we would like to ask you to answer a few socio-demographic questions about yourself. Your answers to these questions will be very useful for analytical purposes.

What is your **gender**?

- ◯ Male
- ◯ Female
- ◯ Other
- ◯ I prefer not to say

What is your **year of birth**?

____

In which **country** do you **currently reside**?

____

World Health Organization

Make Listening Safe

What is the **highest level of education** you have **completed**?

- ◯ Less than High School
- ◯ High School / GED
- ◯ Some College
- ◯ 2-year College Degree
- ◯ 4-year College Degree
- ◯ Masters Degree
- ◯ Doctoral Degree
- ◯ Professional Degree (JD, MD)
- ◯ I prefer not to say

Your answers have been recorded. Thank you very much for taking part to this survey. For more information about the "WHO Safe Listening Initiative" visit https://www.who.int/activities/making-listening-safe
